# Infection-related hospitalization after intensive immunosuppressive therapy among lupus nephritis and ANCA glomerulonephritis patients

**DOI:** 10.1080/0886022X.2020.1763400

**Published:** 2020-05-14

**Authors:** Peihong Yin, Jianbo Li, Qiong Wen, Yagui Qiu, Wenyi Liang, Junxian Wang, Jing Yu, Zhong Zhong, Xiao Yang, Xueqing Yu, Qing Ye, Fengxian Huang

**Affiliations:** a Department of Nephrology, The First Affiliated Hospital, Sun Yat-sen University, Guangzhou, China; b Department of Nephrology, Zhongshan City People’s Hospital, Zhongshan, China

**Keywords:** Lupus nephritis, ANCA glomerulonephritis, infection, hospitalization, immunosuppression

## Abstract

**Introduction:**

This study aimed to investigate the clinical characteristics, risk factors, and outcomes of infection-related hospitalization (IRH) in patients with lupus nephritis (LN) and ANCA glomerulonephritis after intensive immunosuppressive therapy.

**Methods:**

Patients diagnosed with LN or ANCA glomerulonephritis who received intensive immunosuppressive therapy at the First Affiliated Hospital of Sun Yat-sen University from 2005 to 2014 were enrolled. Demographics, laboratory parameters, immunosuppressive agents, and IRH details were collected. Multivariable Cox regression was used, and hazard ratios (HRs) and 95% confidence intervals (CIs) were reported.

**Results:**

Totally, 872 patients with 806 LN and 66 ANCA glomerulonephritis were enrolled, and 304 (34.9%) patients with 433 episodes of IRH were recorded. ANCA glomerulonephritis patients were more vulnerable to IRH than LN patients (53.0% vs. 33.4%, *p* = .001). Multivariable Cox regression analysis showed that ANCA glomerulonephritis (HR = 1.62, 95% CI: 1.06–2.49, *p* = .027), diabetes (HR = 1.82, 95% CI: 1.03–3.22, *p* = .039) and a higher initial dose of prednisone (HR = 1.01, 95% CI: 1.00–1.02, *p* = .013) were associated with a higher likelihood of IRH. Higher albumin (HR = 0.96, 95% CI: 0.94–0.98, *p* < .001), globulin (HR = 0.98, 95% CI: 0.96–0.99, *p* = .008), and eGFR (HR = 0.99, 95% CI: 0.99–1.00, *p* < .001), were associated with a lower likelihood of IRH. The rates of transfer to ICU and mortality for ANCA glomerulonephritis patients were higher than those for LN patients (22.9% vs. 1.9%, *p* < .001, and 20.0% vs. 0.7%, *p* < .001, respectively).

**Conclusions:**

ANCA glomerulonephritis patients had a higher risk of IRH and poorer outcome once infected after intensive immunosuppressive therapy than LN patients. More strict control for infection risks is required for ANCA glomerulonephritis patients who undergo intensive immunosuppressive therapy.

## Introduction

Both systemic lupus erythematosus (SLE) and ANCA vasculitis frequently involve the kidney [1–3]. A study from the southeastern United States involving 21,374 patients with any form of glomerular disease identified in renal biopsy specimens showed that the proportions of lupus nephritis (LN) and ANCA glomerulonephritis were 12.5% and 7.9%, respectively [4]. Overall, 10% of LN and 26.6% of ANCA glomerulonephritis would progress to end-stage renal disease (ESRD) [5,6].

According to the Kidney Disease Improving Global Outcomes (KDIGO) and European Vasculitis Study Group (EUVAS) guidelines, LN and ANCA glomerulonephritis share a similar treatment regimen [7,8], that is, intensive immunosuppressive therapy, which always includes glucocorticoid and immunosuppressive agents. Undoubtedly, the risk of treatment-related infection is increased due to the combined use of glucocorticoid and immunosuppressive agents, which would inhibit the immune function of the patients [9–12]. Moreover, infection among LN and ANCA glomerulonephritis patients was suggested to be a leading cause of rehospitalization and mortality and was associated with a significant economic and social burden [13–17]. In clinical practice, we noticed that the infection rates seemed to be higher and the outcomes seemed to be poorer in patients with ANCA glomerulonephritis than in patients with LN after intensive immunosuppressive therapy, but this observation had not been rigorously investigated.

Thus, we conducted this retrospective study to evaluate the clinical characteristics, risk factors, and outcomes of infection-related hospitalization (IRH) among these patients after intensive immunosuppressive therapy.

## Methods

### Materials

This was a retrospective study. All inpatients diagnosed with LN or ANCA glomerulonephritis at the First Affiliated Hospital of Sun Yat-sen University from 1 January 2005 to 31 December 2014 were screened. All of the selected patients fulfilled the following inclusion criteria: ① age ≥18 years; ② intensive immunosuppressive therapy initiated at our hospital; and ③ regular follow-up. Intensive immunosuppressive therapy was defined as therapy with glucocorticoids and immunosuppressants [18]. Regular follow-up means that patients visited specialist physicians regularly every 1–3 months. Patients who had already received immunosuppressive therapy at another medical institution before admission to our hospital, were in poor compliance, or had a history of renal replacement therapy were excluded. Poor compliance was defined when medical records (electrical or manual) were unavailable due to telephone-confirmed irregular follow-up of patients. All LN patients fulfilled the 1997 revised SLE classification criteria of the American College of Rheumatology with renal damage [19]. All ANCA glomerulonephritis patients met the criteria for ANCA-associated vasculitis outlined by the Chapel Hill Consensus Conference; ANCA glomerulonephritis patients with secondary SLE, rheumatoid arthritis, anaphylactoid purpura, drug abuse, or infection were excluded from the analysis [20]. This study was approved by the ethics committee of the First Affiliated Hospital of Sun Yat-sen University. Informed consent was waived by the committee because of the retrospective nature of the study.

### Data collection

Demographic data including sex, age, primary renal disease, and comorbidities (diabetes, hepatitis, cytomegalovirus disease, and tuberculosis) were collected. Baseline laboratory data before immunosuppressive therapy were also collected, including leukocyte, neutrophil, lymphocyte, and monocyte counts; hemoglobin, albumin, globulin, creatinine, and uric acid levels; and eGFR (calculated with the Chronic Kidney Disease Epidemiology Collaboration (CKD-EPI) equation) [21]. Therapeutic data included the initial dose of glucocorticoid and immunosuppressive agents. The immunosuppressive agents were limited to cyclophosphamide (CTX) and mycophenolate mofetil (MMF) because calcineurin inhibitors (CNIs) were not used in patients with ANCA glomerulonephritis, and other immunosuppressive agents, such as methotrexate (MTX), azathioprine (AZA), rituximab (RTX), and leflunomide, were rarely used in LN and ANCA glomerulonephritis patients at our center. The choice of immunosuppressive agent was mainly made according to the suggestion of the physician. We did not routinely use prophylactic treatment (e.g., trimethoprim/sulfamethoxazole) during immunosuppressive therapy. The infection parameters included the site, type, and severity of infection. Adverse clinical outcomes included transfer to the intensive care unit (ICU) and/or death [22].

IRH details were also collected and analyzed. In our study, recorded infection implied the administration of an antibiotic for the observation of a clinical, microbiological, or radiological suspected infection [23]. An IRH was defined as an event in which infection was a primary or secondary reason for hospitalization [24]. Severe infection was identified according to the ICD-9 codes for bacterial infections (pneumonia, endocarditis, cellulitis, bacteremia, pyelonephritis, septic arthritis, osteomyelitis, and listeriosis), mycobacterial infections, viral infections (herpes zoster, cytomegalovirus, varicella zoster, and influenza), and fungal infections (pneumocystosis, aspergillosis, cryptococcosis, and histoplasmosis) [25]. The determination of pathogens was based on clinical manifestation, therapeutic efficacy of antibiotic agents, etiological examination, or image-based diagnosis [25].

### Statistical analysis

Continuous variables are presented as the mean ± the standard deviation (SD) for normal distributions and the median (interquartile range, IQR) for skewed distributions. Categorical variables are expressed as frequencies and percentages. The *t*-test and Wilcoxon’s rank sum test were employed for comparisons of normal and skewed continuous variable distributions, respectively. The chi-squared test was used for comparisons of categorical variables. We used a multivariable Cox regression model to identify the independent risk factors associated with IRH. Variables with *p* < .1 in univariate analysis and clinical significance were included in the multivariate analysis. The results were expressed as hazard ratios (HRs) with 95% confidence intervals (CIs). *p* Values < .05 were considered statistically significant. Statistical analysis was performed using SPSS version 19.0 (SPSS Inc., Chicago, IL).

## Results

### Demographic, clinical and laboratory characteristics of the study cohort

A total of 872 patients (age: 34.2 ± 12.6 years, male: 17.3%) with 806 patients with LN and 66 with ANCA glomerulonephritis were enrolled in the study. The demographic and clinical characteristics of patients with LN and ANCA glomerulonephritis are displayed in Table 1. Compared with ANCA glomerulonephritis patients, patients with LN were younger (32.9 ± 11.4 years old vs. 49.6 ± 16.1 years old, *p* < .001); presented with a lower proportion of males (14.8% vs. 48.5%, *p* < .001) and a lower proportion of patients with histories of diabetes (1.6% vs. 10.6%, *p* < .001) and tuberculosis (1.1% vs. 9.1%, *p* < .001); presented with lower levels of leukocytes (7.3 ± 4.1 × 10^9^/L vs. 9.0 ± 2.8 × 10^9^/L, *p* = .001), neutrophils (5.4 ± 3.6 × 10^9^/L vs. 7.0 ± 2.5 × 10^9^/L, *p* < .001), albumin (25.9 ± 6.5 g/L vs. 33.3 ± 3.9 g/L, *p* < .001), and serum creatinine [68.0 (51.2, 119.9) μmol/L vs. 288.5 (150.0, 382.3) μmol/L, *p* < .001]; and presented with higher levels of eGFR [97.0 (59.9, 120.1) mL/min/1.73 m^2^ vs. 18.1 (11.7, 41.1) mL/min/1.73 m^2^, *p* < .001] and initial doses of prednisone (51.5 ± 12.6 mg/d vs. 44.9 ± 15.4 mg/d, *p* < .001). However, no significant differences were found in the histories of hepatitis and cytomegalovirus infection; the lymphocyte and monocyte count; or the hemoglobin, globulin, and uric acid levels among these two groups of patients. The choices of immunosuppressive agents were similar between the two groups.

**Table 1.** Demographic and clinical characteristics of patients with lupus nephritis and ANCA glomerulonephritis.

Parameters	Total *n* = 872	Lupus nephritis *n* = 806	ANCA glomerulonephritis *n* = 66	*p* Value
Age (years)	34.2 ± 12.6	32.9 ± 11.4	49.6 ± 16.1	<.001
Male, *n* (%)	151 (17.3)	119 (14.8)	32 (48.5)	<.001
With at least one episode of IRH, *n* (%)	304 (34.9)	269 (33.4)	35 (53.0)	.001
Previous history, *n* (%)				
With diabetes	20 (2.3)	13 (1.6)	7 (10.6)	<.001
With hepatitis	11 (1.3)	10 (1.2)	1 (1.5)	.852
With cytomegalovirus infection	6 (0.7)	4 (0.5)	2 (3.0)	.067
With tuberculosis history	15 (1.7)	9 (1.1)	6 (9.1)	<.001
Leukocyte (×10^9^/L)	7.5 ± 4.0	7.3 ± 4.1	9.0 ± 2.8	.001
Neutrophil (×10^9^/L)	5.6 ± 3.5	5.4 ± 3.6	7.0 ± 2.5	<.001
Lymphocyte (×10^9^/L)	1.4 ± 1.0	1.4 ± 1.1	1.3 ± 0.6	.554
Monocyte (×10^9^/L)	0.4 ± 0.3	0.4 ± 0.3	0.5 ± 0.4	.188
Hemoglobin (g/L)	98.6 ± 23.0	98.8 ± 23.4	95.9 ± 16.6	.321
Albumin (g/L)	26.5 ± 6.6	25.9 ± 6.5	33.3 ± 3.9	<.001
Globulin (g/L)	25.7 ± 8.1	25.7 ± 8.3	25.9 ± 5.6	.836
Serum creatinine (μmol/L)	70.3 (52.3, 136.5)	68.0 (51.2, 119.9)	288.5 (150.0, 382.3)	<.001
eGFR (mL/min/1.73 m^2^)	93.7 (49.9, 118.6)	97.0 (59.9, 120.1)	18.1 (11.7, 41.1)	<.001
Uric acid (μmol/L)	392.9 ± 148.0	391.9 ± 151.1	404.7 ± 102.8	.500
Initial dose of prednisone (mg/d)	51.0 ± 12.9	51.5 ± 12.6	44.9 ± 15.4	<.001
Immunosuppressive agents, *n* (%)				.378
CTX	733 (84.1)	675 (83.8)	58 (87.9)	
MMF	139 (15.9)	131 (16.2)	8 (12.1)	

IRH: infection-related hospitalization; CMV: cytomegalovirus; eGFR: estimated glomerular filtration rate; CTX: cyclophosphamide; MMF: mycophenolate mofetil.

Values were expressed as mean ± SD, median (interquartile range), or number (percentage).

### IRH among patients with LN and ANCA glomerulonephritis after intensive immunosuppressive therapy

**Figure 1.**
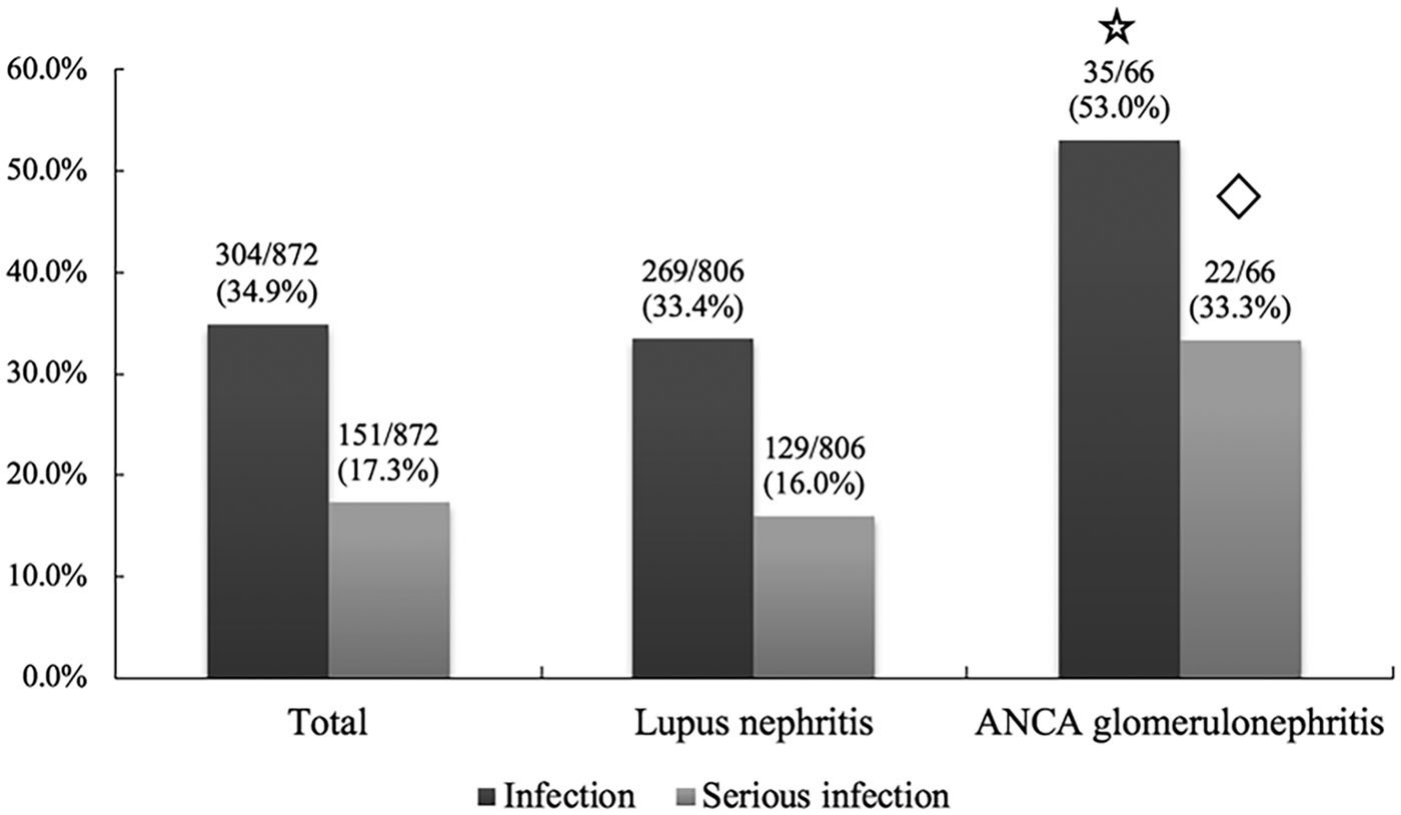
Infection rate of patients after intensive immunosuppressive therapy. Infection rate referred to the percentage of patients with at least one episode of IRH. The serious infection rate referred to the percentage of patients with at least one episode of severe infection. 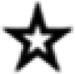
*p*=.001 compared to lupus nephritis; 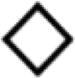
*p*<.001 compared to lupus nephritis.

**Figure 2.**
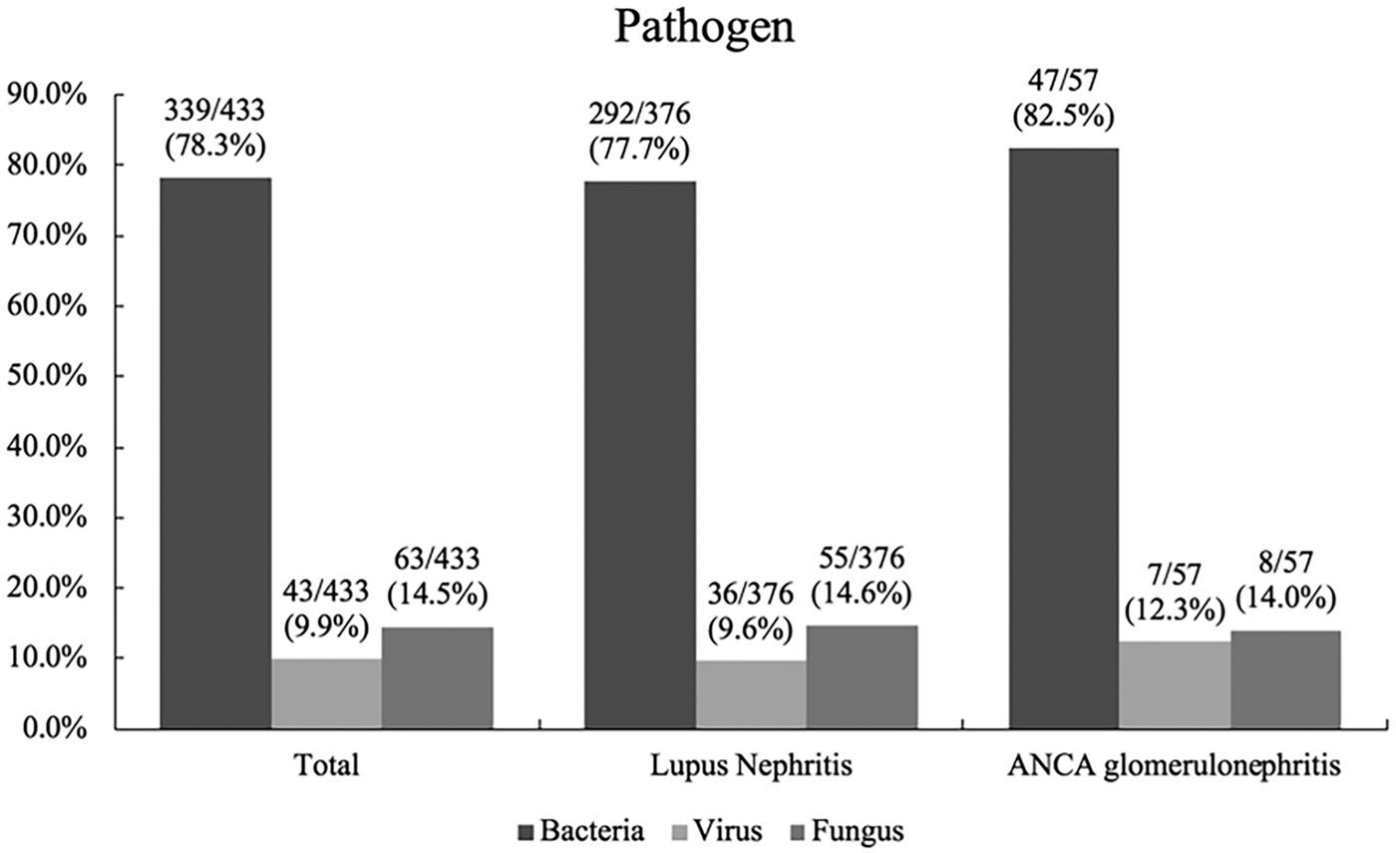
Pathogens of infection for IRH among patients with LN and ANCA glomerulonephritis after intensive immunosuppressive therapy. The determination of pathogen was based on clinical manifestation, therapeutic efficacy of antibiotic agents, etiological examination, or image-based diagnosis. Lupus nephritis vs. ANCA glomerulonephritis: bacteria: *p* = .413; virus: *p* = .524; fungus: *p* = .906, respectively. IRH: infection-related hospitalization.

In total, there were 304 patients (34.9%) who had experienced at least one episode of IRH, and 151 patients (17.3%) had experienced at least one episode of severe infection. A total of 433 episodes of IRH and 201 (46.4%) episodes of severe infection were observed. The average follow-up time was 16.5 (13.9, 22.8) months. The median time from the beginning of intensive immunosuppressive treatment to the first episode of IRH in LN patients and ANCA glomerulonephritis patients was 1 (1, 2) month and 2 (1, 4) months, respectively. Notably, the rates of IRH and severe infection in ANCA glomerulonephritis patients were significantly higher than those in LN patients (53.0% vs. 33.4%, *p* = .001 and 33.3% vs. 16.0%, *p* < .001, respectively), as shown in Figure 1. The comparison of IRH rates in different time periods is displayed in Table 2. IRH rates after 6 months in patients with ANCA glomerulonephritis were significantly higher than those in patients with LN. For all episodes of IRH, bacteria were the most common pathogen (78.3%), as shown in Figure 2. The most common site of infection was the respiratory system (67.0%), followed by skin and soft tissue (17.3%), the urinary system (13.6%) and the digestive system (6.2%). There was no statistically significant difference in infection site between patients with LN and patients with ANCA glomerulonephritis (Table 3).

**Table 2.** Comparison of IRH rates in different time periods.

	Total *n* = 872	Lupus nephritis *n* = 806	ANCA glomerulonephritis *n* = 66	*p* Value
3 months^a^, *n* (%)	244 (28.0)	219 (27.2)	25 (37.9)	.062
6 months^b^, *n* (%)	276 (31.7)	247 (30.6)	29 (43.9)	.026
12 months^c^, *n* (%)	290 (33.3)	258 (32.0)	32 (48.5)	.006
24 months^d^, *n* (%)	296 (33.9)	262 (32.5)	34 (51.5)	.002

IRH: infection-related hospitalization.

^a^ The rate of at least one episode of IRH during 3 months after intensive immunosuppressive therapy.

^b^ The rate of at least one episode of IRH during 6 months after intensive immunosuppressive therapy.

^c^ The rate of at least one episode of IRH during 12 months after intensive immunosuppressive therapy.

^d^ The rate of at least one episode of IRH during 24 months after intensive immunosuppressive therapy.

**Table 3.** The infection sites of patients with IRH after intensive immunosuppressive therapy.

Infection sites	Total (*n* = 433)	Lupus nephritis (*n* = 376)	ANCA glomerulonephritis (*n* = 57)	*p* Value
Respiratory system, *n* (%)	290 (67.0)	247 (65.7)	43 (75.4)	.191
Skin and soft tissue, *n* (%)	75 (17.3)	69 (18.4)	6 (10.5)	.205
Digestive system *n*, (%)	27 (6.2)	20 (5.3)	7 (12.3)	.066
Urinary system, *n* (%)	59 (13.6)	56 (14.9)	3 (5.3)	.077
Central nervous system, *n* (%)	2 (0.5)	2 (0.5)	0	.452
Blood, *n* (%)	14 (3.2)	13 (3.5)	1 (1.8)	.464
Others, *n* (%)	7 (1.6)	6 (1.6)	1 (1.8)	.930

IRH: infection-related hospitalization.

**Figure 3.**
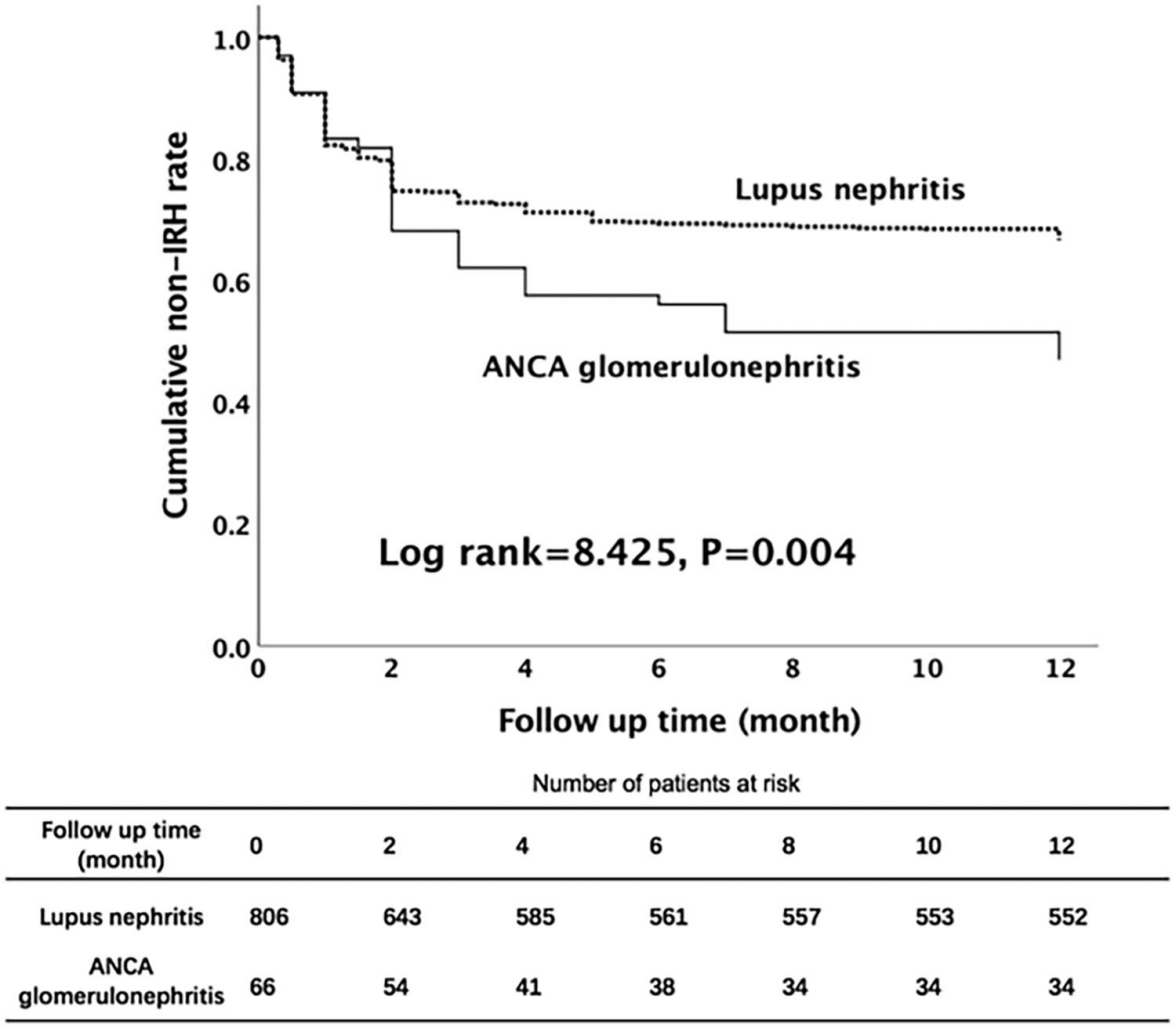
Survival curve and log rank test analysis for first-year IRH among patients with autoimmune renal diseases after intensive immunosuppressive therapy. IRH: infection-related hospitalization.

When Kaplan–Meier’s curves were plotted for cumulative first-year IRH rate, there was a significant difference between patients with LN and patients with ANCA glomerulonephritis (log rank *p* = .004, Figure 3).

### Risk factors for first-year IRH in patients with LN and ANCA glomerulonephritis after intensive immunosuppressive therapy

Cox regression analyses were performed to assess the risk factors for IRH after intensive immunosuppressive therapy in patients with LN and ANCA glomerulonephritis (Table 4). Multivariable Cox regression analysis showed that ANCA glomerulonephritis (HR = 1.62, 95% CI: 1.06–2.49, *p* = .027), diabetes (HR = 1.82, 95% CI: 1.03-3.22, *p* = .039), and a higher initial dose of prednisone (HR = 1.01, 95% CI: 1.00–1.02, *p* = .013) were associated with a higher likelihood of IRH. Higher laboratory values, including albumin (HR = 0.96, 95% CI: 0.94–0.98, *p* < .001) and globulin (HR = 0.98, 95% CI: 0.96–0.99, *p* = .008) levels and eGFR (HR = 0.99, 95% CI: 0.99–1.00, *p* < .001), were associated with a lower likelihood of IRH.

**Table 4.** Risk factors associated with IRH.

Parameters	Univariate Cox regression		Multivariable Cox regression	
	HR (95% CI)	*p* Value	HR (95% CI)	*p* Value
Age (per 1-year interval)	1.01 (1.00, 1.02)	.016	1.00 (0.99, 1.01)	.990
Gender (male vs. female)	1.24 (0.93, 1.64)	.137		
Primary kidney diseases				
Lupus nephritis	1 (reference)		1 (reference)	
ANCA glomerulonephritis	1.65 (1.16, 2.35)	.005	1.62 (1.06, 2.49)	.027
Previous history				
With diabetes	2.07 (1.19, 3.61)	.010	1.82 (1.03, 3.22)	.039
With hepatitis	0.77 (0.25, 2.39)	.644		
With cytomegalovirus infection	2.13 (0.79, 5.70)	.134		
With tuberculosis history	1.42 (0.67, 3.01)	.356		
Leukocyte (per 1.0 × 10^9^/L)	1.01 (0.98, 1.04)	.589		
Neutrophil (per 1.0 × 10^9^/L)	1.02 (0.99, 1.05)	.286		
Lymphocyte (per 1.0 × 10^9^/L)	0.91 (0.81, 1.02)	.088	1.02 (0.90, 1.15)	.767
Hemoglobin (per 1.0 g/L)	1.00 (0.99, 1.00)	.167		
Albumin (per 1.0 g/L)	0.95 (0.94, 0.98)	<.001	0.96 (0.94, 0.98)	<.001
Globulin (per 1.0 g/L)	0.96 (0.95, 0.98)	<.001	0.98 (0.96, 0.99)	.008
Serum creatinine (per 1.0 μmol/L)	1.00 (1.00, 1.00)	<.001		
eGFR (per 1.0 mL/min/1.73 m^2^)	0.99 (0.99, 0.99)	<.001	0.99 (0.99, 1.00)	<.001
Uric acid (per 1.0 μmol/L)	1.00 (1.00, 1.00)	<.001		
Initial dose of prednisone (every 1 mg/d)	1.01 (1.00, 1.02)	.015	1.01 (1.00, 1.02)	.013
Immunosuppressive agents				
CTX	1 (reference)			
MMF	0.98 (0.72, 1.33)	.890		

IRH: infection-related hospitalization; eGFR: estimated glomerular filtration rate; CTX: cyclophosphamide; MMF: mycophenolate mofetil.

Variables with *p* < .1 in univariate analysis and clinical significance were included in multivariable analysis.

### Adverse outcome of first-year IRH in patients with LN and ANCA glomerulonephritis after intensive immunosuppressive therapy

Thirteen (8.6%) patients needed to be transferred to the ICU for further therapy during severe infection, and 11 (7.3%) died. The rates of transfer to the ICU and mortality for patients with ANCA glomerulonephritis were significantly higher than those with LN (36.4% vs. 3.9%, *p* < .001, and 31.8% vs. 3.1%, *p* < .001, respectively), as displayed in Table 5. Cases of the patients who died because of infection are shown in Table 6.

**Table 5.** Comparison of clinical outcome of severe infection according to primary diseases.

	Total (*n* = 151)	Lupus nephritis (*n* = 129)	ANCA glomerulonephritis (*n* = 22)	*p* Value
Transferred to ICU, *n* (%)	13 (8.6)	5 (3.9)	8 (36.4)	<.001
Dead caused by infection, *n* (%)	11 (7.3)	4 (3.1)	7 (31.8)	<.001

IRH: infection-related hospitalization.

**Table 6.** Cases of the patients who died because of infection.

Patients number	Primary disease	Type of infection	Infection site	Immunosuppressive agent	Course of immunosuppressive therapy (months)
1	Lupus nephritis	Bacteria	Respiratory system and blood	CTX	2
2	Lupus nephritis	Bacteria and virus	Skin and soft tissue and blood	CTX	1
3	Lupus nephritis	Bacteria	Respiratory system	CTX	2
4	Lupus nephritis	Bacteria and fungus	Respiratory system	CTX	3
5	ANCA glomerulonephritis	Bacteria	Respiratory system and blood	CTX	2
6	ANCA glomerulonephritis	Bacteria and fungus	Respiratory system	CTX	2
7	ANCA glomerulonephritis	Bacteria and virus and fungus	Respiratory system	MMF	2
8	ANCA glomerulonephritis	Bacteria	Respiratory system	MMF	3
9	ANCA glomerulonephritis	Bacteria and fungus	Respiratory system	CTX	4
10	ANCA glomerulonephritis	Bacteria and fungus	Respiratory system	CTX	2
11	ANCA glomerulonephritis	Bacteria	Respiratory system	MMF	3

CTX: cyclophosphamide; MMF: mycophenolate mofetil.

## Discussion

Our study investigated the clinical characteristics, risk factors, and outcomes of IRH in patients with LN and ANCA glomerulonephritis after intensive immunosuppressive therapy. We mainly demonstrated that the rate of IRH in ANCA glomerulonephritis patients was significantly higher than that in LN patients after intensive immunosuppressive therapy. Additionally, we found that the former had poorer outcomes after infection.

Undoubtedly, the combined use of glucocorticoid and immunosuppressive agents has increased the rate of remission in patients with LN and ANCA glomerulonephritis, resulting in a prolonged renal survival rate and a lower mortality rate [11,14,26]. However, the risk of treatment-related infection increased simultaneously [10,11,14,23]. It was reported that the incidence rate of overall infection in LN patients was 23.9 per 100 person-years [14], and the serious infection rates were 8.2–50 per 100 patient-years for these patients [27]. In addition, the infection rate became even higher with the usage of glucocorticoids and immunosuppressive agents [14,28]. Goupil et al. reported that the infection rate of ANCA-associated vasculitis was 53% [23], and other studies indicated that the rate of infection requiring admission to the hospital in these patients was 21.9–46.2% after intensive immunosuppressive therapy [16,17,29,30]. In the current study, we found that 33.4% of LN patients had IRH after intensive immunosuppressive therapy, similar to the results of previous studies [31–33]. Notably, approximately 16.0% of these patients had serious infections. In addition, we illustrated that 53.0% of patients with ANCA glomerulonephritis suffered from IRH, and 33.3% of them experienced serious infection. Hence, the risk of infection was high for LN patients after intensive immunosuppressive therapy, and this phenomenon was more obvious in patients with ANCA glomerulonephritis. The determination of risk factors that are significantly associated with IRH and guide clinical treatment is of great significance. Our results indicated that most episodes of IRH occurred within the first year after intensive immunosuppressive therapy; therefore, we chose to assess risk factors for first-year IRH.

Our study illustrated several risk factors that were significantly associated with IRH. First, a lower level of baseline albumin was a significant risk factor for IRH. The albumin level is a generally accepted parameter that impacts the clinical outcome of patients with LN and ANCA glomerulonephritis. As reported, a lower level of albumin was significantly associated with infection and mortality among these patients [34,35]. Second, a lower level of globulin also increased the risk of IRH. The degree of globulin was supposed to be a landmark of immune conditions. Reduced levels of globulin refer to losses of immunoglobulin and complement and decrease in circulating T lymphocytes, which can predispose patients to infections [36–38]. We also observed that the initial dose of prednisone was positively associated with IRH. As a higher initial dose of prednisone referred to stronger immunosuppression during the induction period, patients would be more vulnerable to infection [39]. Additionally, we demonstrated that baseline renal function was negatively associated with IRH. As reported, patients with decreased renal function were at increased risk for infection [40], and a worse renal function stage indicated a higher risk of all-cause IRH [24]. Hence, to reduce the risk of IRH, a proper intensive immunosuppressive treatment regimen should be settled on after careful consideration of the aforementioned risk factors.

The most important finding of this study was that ANCA glomerulonephritis was a significant risk factor for IRH among our study population. We demonstrated that the rate of IRH in ANCA glomerulonephritis patients was significantly higher than that in LN patients after intensive immunosuppressive therapy, and we found that the former had poorer outcomes after infection. A possible explanation for these findings might include the following: ① the patients with ANCA glomerulonephritis were significantly older than those with LN in our study. Generally, elderly patients are known to have a relatively weaker immune condition, posing a higher risk of infection and poorer outcome [41]. Although multivariable Cox regression analysis did not show that age was significantly associated with IRH in this study population, we still need to pay close attention to age when settling on a therapy regimen. ② A remarkably lower level of eGFR among ANCA glomerulonephritis patients was found. As discussed above, decreased renal function was a significant risk factor for IRH [24,40]. ③ A history of diabetes was more frequent in ANCA glomerulonephritis patients than in LN patients. It is widely accepted that diabetic patients have an increased propensity to develop infections [42,43], which would probably be a reason for the increasing rate of IRH in patients with ANCA glomerulonephritis. However, a history of diabetes was relatively infrequent in the patients in our study, and there are wide CIs and low statistical power in our study. Thus, whether DM is an independent risk factor for IRH needs further investigation. Interestingly, we also found that patients with ANCA glomerulonephritis had higher levels of baseline albumin and lower initial doses of prednisone than patients with LN. Both variables should decrease the risk of IRH in our study, as discussed above. However, albumin levels could be improved effectively during the course of disease after appropriate therapy, and we supposed that baseline albumin levels might not play a decisive role in the incidence of IRH when compared with age, baseline kidney function and diabetic comorbidities. On the other hand, though the clinicians had decreased the initial dose of prednisone for patients with ANCA glomerulonephritis compared with LN patients, such measures could still not remarkably decrease the rate of IRH for these patients. Thus, a low dose of initial prednisone plus immunosuppressive agents should still be carefully considered for patients with ANCA glomerulonephritis, especially in situations of older age and decreased baseline renal function. Although IRH in ANCA glomerulonephritis patients was more frequent, IRH is still a high burden for LN patients, which indicates that intensive immunosuppressive therapy should be carefully considered in both LN and ANCA glomerulonephritis patients to reduce IRH.

Nevertheless, the present study has some limitations. First, this was a single-center retrospective study. Whether these findings could be generalized to other populations should be explored further. Second, we did not include IRH data from other medical institutions, which would lead to selection bias. However, as one of the most prestigious renal departments in China, patients in our center were in good compliance and would, therefore, be more likely to be hospitalized in our center if infection occurred. In addition, data on renal pathology, whether patients received intravenous hormone pulse therapy or plasma exchange, cumulative dose and duration of prednisone and immunosuppressive agents, and maintenance immunosuppressive treatments were not included in our study. Additionally, the sample size and patterns of our study population were imbalanced. Hence, further studies with multiple centers, larger sample sizes, and more comprehensive baseline parameters are warranted.

## Conclusions

ANCA glomerulonephritis patients had a higher risk of IRH and poorer outcome once infected after intensive immunosuppressive therapy than LN patients. More strict control of infection risk is required for ANCA glomerulonephritis patients who undergo intensive immunosuppressive therapy.
